# Older adults who receive homecare are at increased risk of readmission and mortality following a short ED admission: a nationally register-based cohort study

**DOI:** 10.1186/s12877-021-02644-6

**Published:** 2021-12-15

**Authors:** Mette Elkjær, Donna Lykke Wolff, Jette Primdahl, Christian Backer Mogensen, Mikkel Brabrand, Bibi Gram

**Affiliations:** 1 Department of Emergency Medicine, University hospital of Southern Denmark, Kresten Philipsens Vej 15, 6200 Aabenraa, Denmark; 2 Department of Regional Health Research, University of Southern Denmark, Odense, Denmark; 3 Research Unit of Health Sciences, Hospital of South West Jutland, Esbjerg, University hospital of Southern Denmark, Esbjerg, Denmark; 4 Department of Internal Medicine, University hospital of Southern Denmark, Aabenraa, Denmark; 5 Sygehus Sønderjylland, University hospital of Southern Denmark, Aabenraa, Denmark; 6 Danish Hospital for Rheumatic Diseases, University hospital of Southern Denmark, Sønderborg, Denmark; 7 Department of Emergency Medicine, University hospital of Southern Denmark, Esbjerg, Denmark; 8 Department of Emergency Medicine, Odense University Hospital, Odense, Denmark

**Keywords:** Primary health care, Older adults, Death, Acute illness, Acute hospitalisation, Multimorbidity, Comorbidities, Emergency medicine

## Abstract

**Background:**

Older adults admitted to an emergency department (ED) who are dependent on homecare may be especially challenged with respect to readmission and mortality. This study aimed to assess whether receiving homecare prior admission was associated with readmission or mortality within 30 days of a short ED admission and to explore whether the amount of homecare received was associated with an increased risk of readmission or mortality.

**Methods:**

This nationwide register-based cohort study included patients aged 65 or above who were admitted to an ED at any Danish hospital from 1 December 2016 to 30 November 2017 and discharged within 48 h. Data were extracted from national registers through Statistics Denmark. Homecare was categorized into groups; patients without homecare and three groups according to the amount of homecare received per week. Logistic regression analyses were used to explore the association between the four homecare groups and outcomes, readmissions and mortality.

**Results:**

In total, 80,517 patients (51% female, median age 75 years) were included in the study. Overall, 64,886 patients without homecare, 15,631 (19%) patients received homecare (64% female, median age 83 years), of which 4938 patients received homecare ≤30 min, 4033 received > 30 min to ≤120 min and 6660 received > 120 min per week. The risk of readmission and mortality increased concurrently with the minutes of homecare received: Patients receiving homecare > 120 min per week had the highest odds ratios (ORs) for readmission within 30 days (OR 1.8 95% CI: 1.7–1.9) and mortality within 30 days (OR 4.5 95% CI: 4.1–4.9) compared with patients without homecare.

**Conclusion:**

Receiving homecare was associated with an increased risk of readmission and death following a short ED admission. Collaboration between the ED and primary health care sector in relation to rehabilitation and end-of-life care is essential to improve quality of care for older adults who receive homecare, particularly those receiving homecare > 2 h a week, because of their increased risk of readmission and mortality.

**Supplementary Information:**

The online version contains supplementary material available at 10.1186/s12877-021-02644-6.

## Background

Admission at emergency department (ED) is especially challenging for older adults aged 65 or above owing to a high prevalence of multimorbidity [1–3]. One-fifth of all acutely admitted older adults are readmitted within 30 days, leading to a significant financial burden for society [4]. Moreover, an acute admission is associated with a critical increase in risk of mortality [5]. Among acutely admitted older adults aged 65 or above who are discharged, 20% will die within 1 year of discharge [5]. Although considerable research has been conducted on older adults who are acutely admitted, there is a lack of agreement on how to identify patients at risk of readmission and mortality.

A 2015 study from Belgium reported that older adult patients 75 years or above who were discharged from an ED had a higher readmission rate compared with other hospitalised patients [3]. ED care involves high activity, and the accelerated treatment processes and rapid discharges might affect the readmission rate. A Danish study concluded that 49% of readmissions were preventable among older adults 75 years or above and these were more likely to occur soon after hospital discharge [6]. In addition, a study from the Netherlands found that (adults median aged 71) the most common reason for preventable readmission was a lack of coordination within the health care system [7]. Identifying patients at risk for readmission would allow health professionals to apply target interventions to prevent readmissions among the patients with the greatest needs.

Acutely admitted older adults are often extraordinarily fragile with multiple comorbidities, functional limitations and cognitive impairments [1, 2, 8]. Municipalities offer cost-free homecare to residents in Denmark for those who find activities of daily living (ADL) challenging because of functional limitations or cognitive impairments [9]. The probability of receiving homecare increases with age, and two-thirds of those who receive homecare are aged 65 or above [10]. In 2015, around 12% of older adults aged 65 or above received homecare in Denmark [9].

Around 35% of medical patients in a Danish ED aged 65 or above, receive homecare [11]. A population-based cohort study from Denmark included 3775 medical patients from a single municipality and reported that receiving homecare before an acute admission was a strong predictor of subsequent readmission and mortality [11]. However, it is not known whether these results are transferable to an ED population and whether the risk of readmission and mortality is associated with the amount of homecare received.

We hypothesise that the risk of readmission and mortality among older adults receiving homecare is associated with their ADL difficulties, which is reflected in the amount (in time) of homecare they receive per week. We categorise homecare based on the assumption that the majority of patients receiving ≤30 min per week only receive practical help, the majority of patients receiving > 30 to ≤120 min per week receive some assistance with personal care in addition to practical help and the majority of the patients receiving > 120 min of homecare are dependent on daily assistance for ADL. Thus, we hypothesise that the amount of help received reflects the degree of dependency on external help.

Examination of whether older adults receive homecare at the time of admission to the ED may be a simple way to identify patients at high risk of readmission or mortality. Thus, this study aimed to assess whether receiving homecare was associated with readmission or mortality within 30 days in older adults, after a short ED admission, and to explore whether the amount of homecare received was related to an increased risk of readmission or mortality.

## Methods

### Study design and setting

A retrospective register-based cohort study was conducted. National data on acutely admitted patients were extracted from Danish registers to construct a database on readmission rates, mortality and associated variables. The Danish health care system is tax funded for all 5.8 million residents, and Danish patients do not bear any additional financial costs for hospital care and treatment or for homecare.

The Danish EDs are organised with hospital beds for patients in need of a short admission and where the discharge is planned directly from the ED. A short ED admission is nationally defined as an admission less than 48 h.

Homecare to support ADL consists of “personal care”, which includes assistance with personal hygiene, dressing, morning routines and mealtimes, and “practical help”, which includes assistance with cleaning, laundry and shopping. The type and amount of homecare allocated to a resident is based on an individual assessment, with the specific aim of restoring, maintaining and improving mental and physical functionality [9]. Individual needs are continually adjusted and are planned by a specialist. Data on the type (personal/practical) and amount of homecare are shared electronically between the municipality of residence and local and regional hospitals at admission and discharge.

### Data sources

Data were extracted from the following registries in the Statistics Denmark [12].

The Danish Civil Registration System (CRS) contains individual-level information on all persons residing in Denmark [13]. The unique 10-digit identification number assigned to all Danish citizens in the CRS at birth or immigration enables linking data from different data sources. We extracted information about marital status, age, sex and the date of death among the study population from this database.

The Danish National Patient Registry (DNPR) possesses longitudinal data on inpatient and outpatient contacts, as well as discharge diagnosis codes according to the International Classification of Diseases, Revision 10 (ICD10) [14]. The DNPR was used to define the study population and obtain information about readmission and comorbidity.

The Danish Registration of Elderly contains information about services supplied by the municipalities to residents. Information about health care use and homecare services among acutely admitted older adult patients was extracted [15].

The Danish National Prescription Registry (NPR) contains individual-level data on prescriptions dispensed from pharmacies [16]. Information about the prescription of medication for mental health Anatomical Therapeutic Chemical (ATC) codes (N05–N07) was extracted and used to describe mental comorbidity.

The Income Statistics Register provides data on the income composition of the entire Danish population. The patients’ disposable income was extracted from the Income Statistics Register [17].

### Study population

From 1 December 2016 to 30 November 2017, patients aged 65 years or above with an admission to any Danish ED were included in the study population. The study population was extracted from the DNPR. In the DNPR, patients are registered as inpatients or outpatients (patient type) and as admission type, acute or planned. As a proxy for ED admissions, we included all acute inpatients (admissions) with a length of stay below 48 h in the study population.

Since each medical specialty, department or hospital contact is coded as an individual contact in the DNPR, contacts of less than 3 h apart were merged into a single contact. An additional file shows this in more detail (see Additional file 1). We excluded patients registered as an acute outpatient visit only, patients living in residential care and patients without Danish residency. The patient’s first contact at the hospital in the study period was registered as the index admission. The follow-up periods for the study population were 30 days regarding readmission and 360 days regarding mortality.

### Outcome variables

The primary outcome was readmission, which was defined as an acute contact, occurring within 30 days of the day of previous discharge. We included acute outpatient contacts above 6 h equal to inpatients (admissions) as the primary outcome as we do not consider them to be outpatient appointments. Secondary outcomes were readmission within 7 days and mortality within 30, 180 and 360 days. Primary and secondary outcome variables were assessed from the day of discharge from the index admission.

### Exposure variable

The study population was categorised into one of the following four groups according to the proportion of homecare received: 1) patients without homecare, 2) patients receiving homecare ≤30 min per week, 3) patients receiving homecare between > 30 to ≤120 min per week and 4) patients receiving homecare > 120 min per week.

Homecare is defined as personal and practical help received and recorded in minutes per week. To assess the total time of homecare, we merged time allocated to practical help and time allocated to personal help. The homecare variable was assessed a month before the index admission and was calculated as a mean (minutes) of homecare received per week throughout the entire month. Patients receiving homecare for less than 1 min per week were categorised as patients without homecare.

The Danish Registration of Elderly receives monthly data from the municipalities. Unfortunately, data from some municipalities are inadequate and there may be variations over the year that are not recorded. If the municipalities do not approve the data as valid, Statistics Denmark uses the data delivered the month previously. Statistics Denmark provides information on those months for which data might not be valid. Using this information, we performed a sensitivity analysis (see Appendix B).

### Covariables

Explanatory variables were selected based on their possible influence on readmission and mortality. We used information about the patients’ age, sex and marital status (unmarried/married) recorded on the date of their index admission. Disposable income was included as a socio-economic factor. Disposable income is defined as the amount of money a person has available to spend and save after accounting for income tax. We calculated the median yearly disposable income over the past 5 years up to the index admission. The updated version of the Charlson Comorbidity Index (CCI) [18] was used to calculate and describe the number of patients’ chronic diseases using ICD-10 diagnoses and included both A (primary) and B (secondary) diagnoses. All registered diagnoses from 5 years before the index admission were included. Since the CCI contains little to no information about mental disorders, we used information on medical prescriptions for mental disorders at any time within the year before the index admission as a proxy for mental disorder (yes/no). Different types of medication for mental disorders included antipsychotics, anxiolytics, hypnotics and sedatives, antidepressants, and medication for dementia. Psychostimulants were rarely used (< 0.03%) and were therefore not included. Type of medication was used to describe the cohort and to compare patients receiving homecare with patients not receiving homecare. Type of medication was not included in the analyses.

### Statistical methods

Descriptive statistics were used to report demographic and clinical characteristics for the following categories: all patients, patients without homecare and patients with homecare. Categorical data were reported as frequencies and continuous variables as median and interquartile range (IQR). The distribution of data was assessed with Q-Q plots. Differences between groups were tested with the Wilcoxon rank sum test or Pearson’s chi-square test, depending on the distribution of the data.

The associations between received homecare and outcomes (readmission rate on day 7 and 30 and mortality on day 30, 180 and 360) were tested using logistic regression analyses and presented as crude ORs with 95% confidence intervals (CIs). Furthermore, the regression analyses were preformed adjusting for age, Charlson Comorbidity Index and income (as continuous variables) and sex, marital status and treatment for mental disorder (as categorical variables) and presented as adjusted ORs with 95% CIs. Finally, a margins plot calculating the probability for mortality and readmission within the homecare categories from the adjusted logistic regression analysis was created. Patients who died before readmission were excluded from the analyses calculating the risk of readmission. Further, we repeated all the analyses excluding the months with inadequate data from the municipalities in sensitivity analyses. All analyses were performed using Stata version 16.1 (StataCorp, College Station, TX, USA). *P*-values of < 0.05 were considered statistically significant.

## Results

### Participants

**Fig. 1:**
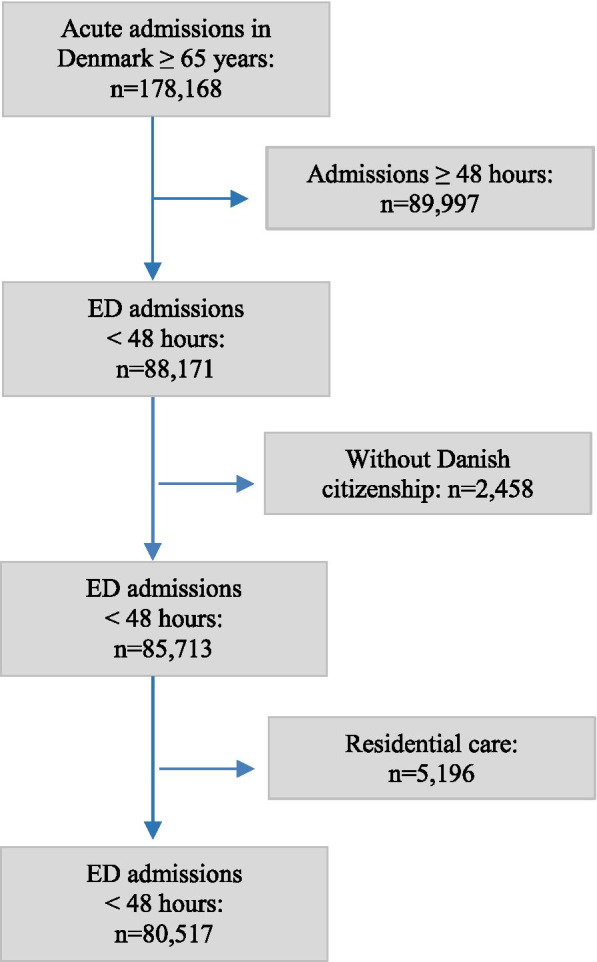
Flowchart of study inclusions and exclusions

In total, 80,517 patients were included as the study population (Fig. 1).

Among the study population, 15,631 patients (19%) received homecare. Patients receiving homecare before the index admission were older (median [IQR]: age 83 [76–88] vs. 74 [70–80]) years, fewer were married (24% vs. 62%), the majority were female (64% vs. 48%), a larger proportion received treatment for mental disorders (53% vs. 30%), and they had a higher CCI score (median [IQR]: 1 [0–2] vs. 0 [0–1]) compared with patients who did not receive homecare (Table 1). Further, patients receiving homecare experienced a significant increased rate of readmission and mortality after an acute admission (Table 1).

**Table 1:** Sociodemographic characteristics of the study population

Variables	All patients ***N*** = 80,517	Patients with no homecare ***n*** = 64,886	Patients receiving homecare ***n*** = 15,631	P-value Homecare vs. no homecare
Aged (year) Median (IQR)	75 (70–82)	74 (70–80)	83 (76–88)	< 0.001
Marital status (married)	43,498 (54%)	39,725 (62%)	3773 (24%)	< 0.001
Sex (female)	40,882 (51%)	30,918 (48%)	9964 (64%)	< 0.001
In treatment for mental disorders	27,517 (34%)	19,243 (30%)	8274 (53%)	< 0.001
In treatment with *				
Antipsychotics	1696 (2%)	1078 (2%)	618 (4%)	< 0.001
Anxiolytics	4311 (5%)	3125 (5%)	1186 (8%)	< 0.001
Hypnotics & sedative	9069 (11%)	6740 (10%)	2329 (15%)	< 0.001
Antidepressants	6706 (8%)	4558 (7%)	2148 (14%)	< 0.001
Medicine for dementia	1272 (2%)	628 (1%)	644 (4%)	< 0.001
Income after taxes (Euro)				
median (IQR)	22,162 (17,756–29,300)	22,323 (17,513–30,435)	21,801 (18,710–25,796)	< 0.001
Charlson Comorbidity Index				
median (IQR)	0 (0–2)	0 (0–1)	1 (0–2)	< 0.001
0	48,548 (60%)	41,358 (64%)	7190 (46%)	
1	10,066 (12%)	7414 (11%)	2652 (17%)	
2	13,562 (17%)	10,418 (16%)	3144 (20%)	
> 3	8341 (11%)	5696 (9%)	2645 (17%)	
Mortality				
< 30 days	3010 (4%)	1790 (3%)	1220 (8%)	< 0.001
< 180 days	7022 (9%)	4320 (7%)	2702 (17%)	< 0.001
< 360 days	10,191 (13%)	6.325 (10%)	3866 (25%)	< 0.001
Readmission				
< 7 days	5371 (7%)	4094 (6%)	1277 (8%)	< 0.001
< 30 days	10,895 (14%)	8127 (13%)	2768 (18%)	< 0.001

Note: IQR is interquartile range and n is numbers of patients. The difference between groups was tested with the Wilcoxon rank sum test (unequal distribution) or Pearson’s chi-squared test. *Patients can appear in more than one category

### Outcomes

The distribution of the amount of homecare was as follows: 4938 patients (32%) received ≤30 min (~ practical help), 4033 patients (26%) received ≥31 and ≤ 120 min (~ personal care in addition to practical help) and 6660 patients (42%) received ≥121 min per week (~ dependent on daily assistance for ADL).

Among the study population, 5371 patients (7%) were readmitted within 7 days and 10,895 patients (14%) were readmitted within 30 days. Table 2 shows the association between amount of homecare received and risk of readmission. The risk of readmission within 30 days increased with the amount of time for which patients received homecare; patients receiving homecare for > 120 min a week had the highest odds for readmission – OR 1.82 (95% CI: 1.70–1.94) – compared with patients without homecare in the crude analysis (Table 2). When adjusting for age, sex, marital status, income, CCI and treatment for mental disorders, patients receiving homecare > 120 min a week still had significant increased odds for readmission – OR 1.43 (95% CI: 1.33–‍1.54) – compared with patients without homecare (Table 2). The increased risk of readmission within 30 days among patients receiving homecare for ≤30 min and between > 30 and ≤ 120 min was similar to that found in the crude analysis.

**Table 2:** Association between homecare categories and readmission

Variables	Patients with no homecare	Patients receiving homecare ≤ 30 min/week	Patients receiving homecare > 30 to ≤120 min/week	Patients receiving homecare > 120 min/week
Readmission < 7 days				
Crude OR (95% CI) Adjusted OR (95% CI)	1 (ref.)	1.26 (1.13–1.40)** 1.15 (1.02–1.29)*	1.26 (1.12–1.42)** 1.13 (1.00–1.29)	1.53 (1.40–1.68) ** 1.30 (1.17–1.43) **
Readmission < 30 days				
Crude OR (95% CI) Adjusted OR (95% CI)	1 (ref.)	1.41 (1.30–1.52) ** 1.22 (1.12–1.31) **	1.46 (1.34–1.59) ** 1.24 (1.13–1.35) **	1.82 (1.70–1.94) ** 1.43 (1.33–1.54) **

Notes: OR is the odds ratio and CI is confidence intervals. The logistic regression analyses were adjusted for age, sex, marital status, income, Charlson Comorbidity Index and treatment for mental disorder. Significance levels with a p-value of < 0.05 are marked with * and significance levels with a *p*-value of < 0.001 are marked with**

In the study population, 3010 (4%) patients died within 30 days, 7022 (9%) within 180 days and 10,191 (13%) within 360 days. Table 3 shows the association between amount of homecare received and mortality. The crude risk for 30-days mortality increased related to the amount of time for which patients received homecare (Table 3). The patients receiving homecare for > 120 min per week had the highest OR for 30-days mortality – OR 4.51 (95% CI: 4.12–4.93) – when comparing the three homecare categories (Table 3).

**Table 3:** Association between homecare categories and mortality

Variables	Patients with no homecare	Patients receiving homecare ≤ 30 min/week	Patients receiving homecare > 30 to ≤120 min/week	Patients receiving homecare > 120 min/week
Mortality < 30 days				
Crude OR (95% CI) Adjusted OR (95% CI)	1 (ref.)	1.72 (1.50–1.99)** 1.08 (0.92–1.26)	2.18 (1.90–2.50)** 1.26 (1.07–1.47)*	4.51 (4.12–4.93)** 2.25 (2.01–2.50)**
Mortality < 180 days				
Crude OR (95% CI) Adjusted OR (95% CI)	1 (ref.)	1.71 (1.56–1.88) ** 1.08 (0.97–1.20)	2.23 (2.03–2.45)** 1.31 (1.18–1.46)**	4.48 (4.20–4.77)** 2.28 (2.11–2.46) **
Mortality < 360 days				
Crude OR (95% CI) Adjusted OR (95% CI)	1 (ref.)	1.81 (1.67–1.96)** 1.14 (1.05–1.25) *	2.34 (2.16–2.54)** 1.39 (1.27–1.52) **	4.71 (4.45–4.99)** 2.45 (2.28–2.62)**

Notes: OR is the odds ratio and CI is confidence intervals. The logistic regression analyses were adjusted for age, sex, marital status, income, Charlson Comorbidity Index and treatment for mental disorder. Significance levels with a p-value of < 0.05 are marked with * and significance levels with a *p*-value of < 0.001 are marked with**

When adjusting for age, sex, marital status, income, CCI and treatment for mental disorders, patients receiving homecare for > 120 min per week still had an increased OR of 2.25 (95% CI: 2.01–2.50) for 30-days mortality compared with patients without homecare (Table 3).

Receiving homecare was associated with an increased probability of readmission and mortality and patient’s receiving homecare > 120 min per week had the highest probability within 30 days from the day of discharge (readmission: 21% (95% CI: 20.1–22.1) and mortality: 11% (95% CI: 10.6–12.1)). For comparison, older adults without homecare had a probability of readmission 13% (95% CI: 12.5–13.1) and of mortality 3% (95% CI: 2.6–2.9) within 30 days.

**Fig. 2:**
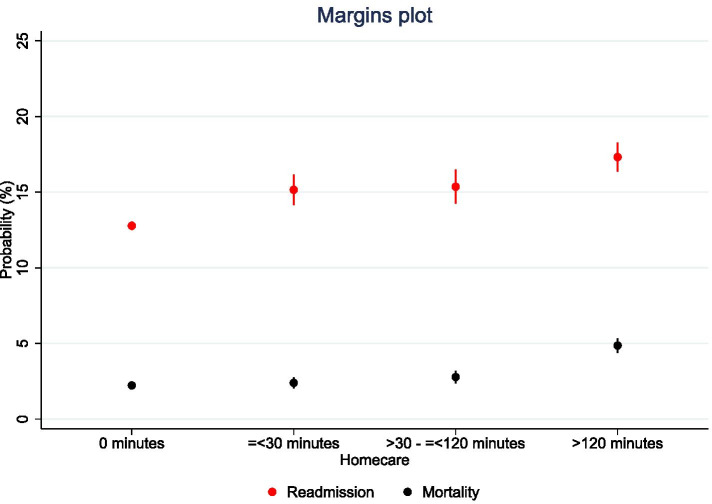
Probability for readmission and mortality within 30 days for the categories of homecare. Note: Results show probability (%) with 95% CI for readmission and mortality within 30 days calculated from the adjusted logistic regression analyses

When adjusting for age, sex, marital status, income, CCI and treatment for mental disorders, patients receiving homecare for > 120 min per week had a probability for readmission within 30 days of 17% (95% CI: 16.3–18.2) and 5% (95% CI: 4.3–5.3) for mortality compared to a probability of readmission of 13% (95% CI: 12.5–13) and 2% (95% CI: 2.1–2.3) of mortality within 30 days among older adults without homecare (Fig. 2).

The sensitivity analyses excluding the months with inadequate data from the municipalities revealed no significance for the results regarding readmission and mortality. An additional file shows this results in detail (see Additional file 2).

## Discussion

Overall, our study showed that older adults aged 65 or above receiving homecare within the month prior to an ED admission were at a significantly higher risk of readmission and a significantly higher risk of dying compared with older adults not receiving homecare. Although we adjusted for explanatory variables such as comorbidity, sex and age, receiving homecare was a strong predictor of readmission and early death. Older adults who were dependent on daily assistance for ADL (receiving homecare for > 2 h) were at the highest risk among the homecare categories.

Among our study population, 19% received homecare, which was fewer than found in other studies [1, 11]; however, our study population differs because it consists of both medical and surgical complaints and solely comprises short ED admissions of < 48 h.

### Readmission and homecare

We found that receiving homecare was a significant predictor of readmission within 30 days of discharge. Older adults receiving homecare > 2 h per week had an increased risk (OR 1.8) corresponding to nearly doubled probability, compared to older adults without homecare (21% vs 13%).

Tanderup et al. also reported that receiving homecare was a strong predictor of readmission among Danish medical patients (≥ 65 years) compared with medical patients without homecare (HR for competing risk 1.8) [11]. In contrast, a study from Belgium of ED patients (≥ 75 years) reported no significant association between readmission and homecare [3]. However, the study from Belgium was relatively small, with only 97 patients [3]. Our study clearly demonstrates that older adult Danish residents receiving homecare are at increased risk of readmission following an ED admission. We adjusted for age, comorbidities, income, sex, marital status and treatment for mental disorders. However, there might be some overlooked confounding variables not included in the analyses affecting to which extent homecare is an independent predictor of an increased risk of readmission.

### Mortality and homecare

We found that receiving homecare was a significant predictor of the risk of dying within 30 days of discharge. Moreover, older adults receiving homecare for more than 2 h a week had a nearly fourfold risk (OR 4.5) and probability (11% vs 3%), compared with patients who did not receive homecare.

Similarly, Tanderup et al. reported that Danish medical patients receiving homecare had an increased risk of mortality within 30 days of an acute admission, with a HR of 2.8, compared with patients not receiving homecare [11]. In addition, we found different risks between the homecare groups. Older adults receiving homecare for > 120 min a week had a significantly higher risk of mortality compared with adults receiving homecare for ≤30 min a week (OR 4.5 vs. OR 1.7). Thus, our study contributes important results to the existing knowledge since older adults dependent on daily assistance for ADL (i.e. receiving homecare for > 2 h) have a markedly increased risk of dying after an ED admission compared to patients without homecare.

A Spanish cohort study identified male sex, comorbidity, hospital admission and degree of pressure ulcers as significant predictors of mortality among older adults receiving homecare [19]. Male sex, age and comorbidity were also identified as independent predictors of one-year mortality in the Danish population of medical patients admitted ≥65 years [11]. We adjusted for age, comorbidities, income, sex, marital status and treatment for mental disorders, and when comparing the crude and the adjusted analyses, the results indicated that some of the selected explanatory variables influenced the results. However, further research is required to elucidate the influence of the covariables (e.g. age, sex and comorbidity).

### Clinical implications

Knowledge about the risk profile of patients receiving homecare can be used to organise and coordinate ED care and preventative treatment. To be able to identify patients at high risk of readmission and death will allow health professionals to implement supportive interventions to ease transfer from the ED to primary health care and prevent unnecessary readmission and early death. Early follow-up visits at home after hospital discharge can reduce preventable readmissions among geriatric patients [6]. Combining several rehabilitative interventions before discharge (individual planning, geriatric assessment and patient education) with follow-up after discharge may further reduce readmissions [20]. In Denmark, information about whether patients receive homecare or not is easy to access from the electronic communication between the municipality and local hospitals, and it is relatively reliable and requires no additional training for use.

A study from Canada found that older adults receiving homecare experienced at least one care transition (moves between care settings such as a home nursing, home and hospital) during the last 30 days before death, and one-fifth experienced two or more hospitalisations [21]. If this knowledge is combined with our results demonstrating a nearly fourfold increased risk of dying within 30 days of discharge among older adults receiving homecare, the ED admission may be considered a time-limited window of opportunity to offer help to fragile patients.

We suggest increased attention given to older adults dependent on daily assistance for ADL (receiving homecare for > 2 h). Care interventions could include evaluation of their needs and preferences for rehabilitative interventions or end-of-life care. Whether older adults receive homecare or not and the amount of time allocated to homecare can also be used to develop a plan for future treatment and care and ensure that collaboration between the ED and primary health care sector focuses on preventative interventions.

### Strengths and limitations

Our study has important strengths. This is the first study to investigate readmission rates after a short ED admission comparing older adult patients related to the amount of homecare received, in a large national cohort. We used the DNPR to identify our study population and linked the patients between the registries using the unique 10-digit identification number. Registry data have high completeness and validity [12–17], and to supplement the CCI, we added data from the Danish NPR to describe the patients’ mental health to obtain a complete picture of the patients’ total comorbidities.

Our study had some limitations. As described, there is no specific registration of ED patients and we thus had to develop a proxy for ED admissions. This coding may have imposed a risk of misclassification bias. However, this data management method is well known and used in other register studies [22, 23]. We included both primary and secondary diagnoses calculating patients CCI but did not differentiate between acute and chronic conditions. Furthermore, lifestyle factors such as smoking and overweight can be confounding factors, but was not possible to included using registry data. Further, we used data on marital status (married/unmarried); thus, we could not tell whether the older adults lived with adult children or cohabitants. However, the study population was aged 65 or above, so we assumed that relatively few persons lived together without being married according Danish culture. Finally, the Danish municipalities had months where data transfer was inadequate due to change of registrations system; however, we completed sensitivity analyses excluding these months, which confirmed the robustness of our results.

## Conclusion

Receiving homecare prior admission was associated with an increased risk of readmission and death following admission to an ED for older adults aged 65 or older. Older adults who received homecare for more than 2 h a week were at the highest risk compared with those who received less or no homecare. Collaboration between the ED and primary health care facility in relation to rehabilitation and end-of-life care is essential to improve the quality of care for older adults who receive homecare, particularly those receiving homecare for more than 2 h a week, because of their increased risk of readmission and mortality.

## Supplementary Information

**Additional file 1.**

**Additional file 2.**

## Data Availability

The data that support the findings of this study are available from Statistics Denmark. However, data were used under licence for the current study and are not publicly available.

## Abbreviations

ED: Emergency department
ADL: Activities of daily living
DNPR: Danish National Patient Registry
NPR: National Prescription Registry
CRS: Danish Civil Registration System
CCI: Charlsons Comorbidity Index
IQR: Interquartile range
CI: Confidence interval
OR: Odds ratio
HR: Hazard ratio
