# Supplementary material for: Older adults who receive homecare are at increased risk of readmission and mortality following a short ED admission: a nationally register-based cohort study
Source: BMC Geriatr. 2021 Dec 15;21:696. doi: 10.1186/s12877-021-02644-6 (PMC8672634; doi:10.1186/s12877-021-02644-6)
Supplement: Supplementary file 1 — Additional file 1. [file 12877_2021_2644_MOESM1_ESM.pdf]

Additional file 1

Definition of emergency department (ED) admission less than 48 hours:

The DNPR includes individual contacts. Patients are registered as one patient contact, an inpatient (A) or outpatient (B) (patient type), and the contact can be acute (1) or non-acute (2) (admission type). If patients are transferred to another medical specialty, department or hospital, this is registered as a new contact. Therefore, an ED admission was defined using the following criteria: the first contact should be acute, the first contact could be registered as both an inpatient either outpatient, but the total admission should include an inpatient registration. These contacts were merged into one admission if there were three hours or less between contacts (see Figure A). Finally, admissions for more than 48 hours were excluded.

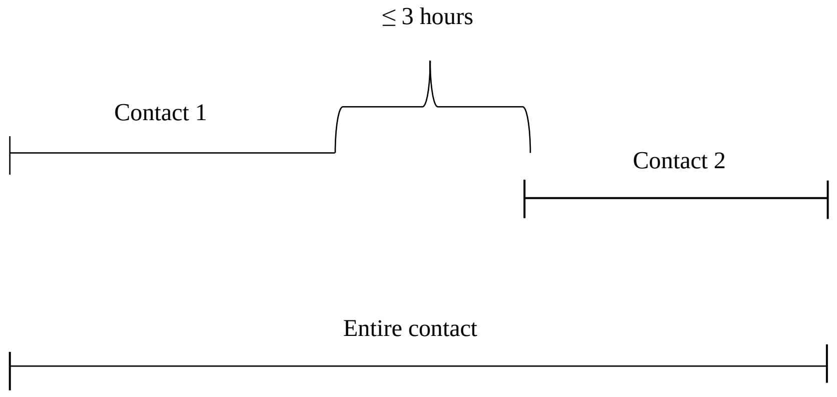

Fig. A: Identification of an acute patient contact

Note: Two contacts from different medical specialties, departments or hospitals were merged and considered one contact if there were three hours or less between two contacts. The figure is inspired by The Danish database for acute and emergency hospital contacts. Clin Epidemiol. 2016; Volume 8:469-74.
