# Supplementary material for: Older adults who receive homecare are at increased risk of readmission and mortality following a short ED admission: a nationally register-based cohort study
Source: BMC Geriatr. 2021 Dec 15;21:696. doi: 10.1186/s12877-021-02644-6 (PMC8672634; doi:10.1186/s12877-021-02644-6)
Supplement: Supplementary file 2 — Additional file 2. [file 12877_2021_2644_MOESM2_ESM.pdf]

Additional file 2

We performed the same analyses, where the months with inadequate data from municipalities were excluded, as sensitivity analyses (7,600 patients) (see Table 4).

Table 4: Association between homecare categories and readmission rate and mortality, excluding patients from municipalities with inadequate data

Variables	Patients with no homecare	Patients receiving homecare ≤ 30 min/week	Patients receiving homecare > 30 to ≤ 120 min/week	Patients receiving homecare > 120 min/week
Readmission < 7days				
Crude OR (95% CI)	1 (ref.)	1.29 (1.16–1.44)**	1.27 (1.13–1.44)**	1.54 (1.40–1.69)**
Adjusted OR (95% CI)		1.18 (1.05–1.33)*	1.15 (1.01–1.30)*	1.31 (1.18–1.45)**
Readmission < 30days				
Crude OR (95% CI)	1 (ref.)	1.44 (1.33–1.56)**	1.48 (1.35–1.62)**	1.85 (1.73–1.98)**
Adjusted OR (95% CI)		1.25 (1.14–1.36)**	1.25 (1.14–1.37)**	1.45 (1.34–1.56)**
Mortality < 30 days				
Crude OR (95% CI)	1 (ref.)	1.80 (1.55–2.07)**	2.27 (1.97–2.62)**	4.67 (4.26–5.12)**
Adjusted OR (95% CI)		1.16 (0.98–1.36)	1.35 (1.14–1.58)**	2.45 (2.18–2.75)**
Mortality < 180 days				
Crude OR (95% CI)	1 (ref.)	1.80 (1.63–1.98)**	2.31 (2.09–2.54)**	4.68 (4.38–5.00)**
Adjusted OR (95% CI)		1.16 (1.04–1.29)*	1.38 (1.24–1.54)**	2.46 (2.27–2.66)**
Mortality < 360 days				
Crude OR (95% CI)	1 (ref.)	1.89 (1.74–2.05)**	2.43 (2.23 – 2.64)**	4.93 (4.65–5.23)**
Adjusted OR (95% CI)		1.22 (1.11–1.34)**	1.46 (1.33 – 1.61)**	2.63 (2.46–2.83)**

Notes: OR is the odds ratio and CI is confidence intervals. The logistic regression analyses were adjusted for age, sex, marital status, income, Charlson Comorbidity Index and treatment for mental disorder. Significance levels with a p-value of < 0.05 are marked with * and significance levels with a p-value of < 0.001 are marked with**.
